# A unified voice to drive global improvements in oral health

**DOI:** 10.1186/s44263-023-00019-0

**Published:** 2023-10-01

**Authors:** Michael Glick, Olivia Urquhart, Ankita S. Bhosale, Alonso Carrasco-Labra, Jaime T. Edelson

**Affiliations:** 1 Center for Integrative Global Oral Health, School of Dental Medicine, University of Pennsylvania, 240 S. 40th Street, 3rd Fl East, Philadelphia, PA USA; 2 Fundación ADM, Institución de Asistencia Privada, Mexico City, Mexico

**Keywords:** Oral health, Stakeholders, Collaboration, Equity, Disabilities, Sustainable development goals, Universal health coverage, Research, Education, Public health initiatives, Workforce

## Abstract

Approximately, 3.47 billion individuals worldwide are affected by oral disorders, with untreated dental caries being the most prevalent issue, impacting 2.30 billion people. Addressing this complex health problem requires a collective and unified approach from the oral health community. However, stakeholders often focus on their own interests, leading to information silos and a lack of diverse perspectives. To foster collaboration, tackle critical topics, and promote effective communication, the Global Oral Health Forum was organized as a 2-day meeting. The forum aimed to dismantle existing silos and cultivate a unified message to drive global improvements in oral health. By bridging gaps between stakeholders and encouraging dialogue, the forum sought to transcend individual interests and foster a holistic approach. The meeting provided a space for diverse viewpoints, concerns, and expertise to converge, facilitating meaningful discussions and overcoming the limitations of an echo chamber. The inclusive environment aimed to integrate different perspectives, generate innovative ideas, and pave the way for impactful changes in oral health worldwide. The outcome of the Global Oral Health Forum I includes suggested action points that emphasize collaboration, knowledge sharing, and policy development. These recommendations aim to promote preventive strategies, improve access to oral healthcare, enhance oral health education, and foster interdisciplinary research and innovation. By implementing these actions, the global oral health community can work together to address the challenges posed by oral disorders and improve oral health outcomes for billions of individuals worldwide.

**Supplementary Information:**

The online version contains supplementary material available at 10.1186/s44263-023-00019-0.

## Background

Approximately, 3.47 billion individuals are believed to be affected by oral disorders [1]. Among these conditions, untreated dental caries (commonly known as tooth decay) in permanent teeth is recognized as the most prevalent health issue, with 2.30 billion affected individuals based on the Global Burden of Disease 2017 report [1]. This health issue cannot be resolved by any single entity within the oral health community alone. It requires a collective and unified approach in order to address effectively.

Stakeholders within the oral health and broader health community often exhibit a tendency to avoid or overlook broader topics, instead focusing solely on advancing specific issues that serve their own interests [2]. Regrettably, these stakeholder groups frequently confine themselves to exclusive gatherings with like-minded individuals, resulting in discussions that resemble an information silo, devoid of diverse perspectives.

Recognizing the need to foster collaboration, address various opinions, tackle critical topics, and promote effective communication, a 2-day meeting, entitled *Global Oral Health Forum I*, was organized on March 30–31, 2023, bringing together multiple stakeholders. The primary objective of this initiative was to dismantle existing silos and cultivate a unified message that would drive global improvements in oral health.

By bridging the gaps between stakeholders and encouraging dialogue, the meeting sought to transcend individual interests and foster a holistic approach to oral health. Through the convergence of diverse viewpoints, concerns, and expertise, the intention was to overcome the limitations of an echo chamber and create a space where meaningful discussions could take place. This inclusive environment aimed to facilitate the integration of different perspectives, generate innovative ideas, and ultimately pave the way for impactful changes in oral health worldwide. The outcome of this meeting is summarized below with suggested action points.

## Executive summary

Interest and concern about the state of global oral health are growing. Documents such as the two-part series on oral health in the *Lancet*, and the World Health Organization’s (WHO) *global oral health status report*, lay out challenges posed by the enormous burden of oral disease around the world — its impact on economies, on the global workforce, and on individuals, particularly the world’s most vulnerable, who live with pain, stigma, and who are unable to participate fully in society [3–5]. Educators and researchers from the dental community have been called to lead the work in organizing an agenda for change [6]. In response to this imperative, the Center for Integrative Global Oral Health at Penn Dental Medicine, University of Pennsylvania, Philadelphia, PA, USA, joined with Fundación ADM, Institución de Asistencia Privada, Mexico City, Mexico, for a 2-day Global Oral Health Forum, March 30–31, 2023, in Merida, Mexico [7]. The purpose of the forum was to provide a platform for stakeholders (Additional file 1) from different sectors of the global oral health community to have an interactive dialogue about prominent issues in oral health. Speakers and panelists were selected based on their affiliation to key stakeholder organizations in oral health, their contribution to the biomedical literature, and expertise in the topic. Conversation was organized around four topics deemed central by the organizing committee and feedback from speakers and panelists to making progress towards the goal of improving oral health for all:

- Equity and disabilities
- United Nations Sustainable Development Goals and universal health coverage
- Research and education
- Public health initiatives and workforce

Every forum participant acknowledged that there has been a general disenfranchisement of oral health and dentistry from general health. Oral health is poorly understood and often not considered by policy makers, resulting in a lack of urgency towards affecting improvements in health outcomes. This lack of understanding is of particular importance since much of the burden of oral disease can be addressed by preventing such disease in the first place. The opening speaker, Sir Michael Marmot, sets the stage for the discussion by referencing his seminal work, *The Health Gap*, which explores the impact of social determinants and income inequality on health [8]. Speaker after speaker noted the stark divide in lived experience concerning oral health, a divide that reflects the impact of poverty, geography, racism, health literacy, culture, and structural economic barriers. Yet, devising and implementing practical solutions that can be recommended as “best buys” require stakeholder consensus, at minimum, that the situation is urgent. Speakers agreed that such consensus can finally be achieved. The oral health community now has the opportunity to avoid some of the mistakes that have often been made in the general medical arena, where costly and resource-intensive Western models of health care, e.g., Bismarck and out-of-pocket models, are proposed as solutions for communities that lack the funds, materials, or workforce to execute the proposed interventions. There was agreement among participants — including educators, policy makers, business leaders, and researchers –— that members of the public must be involved in the dialogue as well as health profession students in dentistry, medicine, nursing, and transdisciplinary fields.

Conversations that bring together stakeholders from all sectors — international, national, and local health organizations, government, education, industry, and healthcare providers — should continue with a praxis-oriented lens, that is, working from an agreed-upon set of globally relevant issues while creating solutions that can be locally implemented in concert with the resources and circumstances of individual countries or communities. The group also affirmed that the global oral health community has the opportunity — and responsibility — to minimize the environmental impact of the oral health delivery system.

## Equity and disabilities

The WHO report on *Health Equity for Persons with Disabilities* notes that an estimated 1.3 billion people — or 16% of the global population — experience a significant disability today [9], indicating that many persons with disabilities die prematurely, have poorer general health, and have inadequate access to care for reasons that are both structural and systemic. Even in amply resourced countries, those responsible for organizing health care have struggled with how best to meet the needs of institutionalized persons with disabilities and even those cared for by their families. The US Centers for Disease Control and Prevention defines disability as any condition of the body or mind (impairment) that makes it more difficult for the person with the condition to participate in daily activities (activity limitation) and interact with the world around them (participation restrictions) [10]. Some people are born with disabilities, but many more people acquire them as a consequence of disease or injury or in relation to aging.

It was suggested by one of the forum participants that poverty and social isolation could also be characterized as disabling since they limit full participation of individuals in society. Speakers applauded the WHO global oral health status report for forcefully bringing the matter of disparities in oral health for persons with disabilities to the attention of policy makers [5]. There is still a paucity of scholarly research on the topic, however, and an even larger gap in the education of health professionals. It was acknowledged that most practicing dentists were never taught how to care for persons with disabilities, particularly adults with disabilities. Dentists who care for patients with disabilities will note that these patients often require more time than a traditional dental visit allows, and providers are generally poorly reimbursed for their work. There was broad consensus that compensation models must change if more providers are going to be recruited to participate in caring for persons with disabilities. Here again, acknowledgement of the critical role of oral health promotion and prevention should be impressed upon policy makers as a natural cost-savings opportunity. It was suggested that payers (i.e., private and government insurers) must be acquainted with the issues and become part of the solution. It was noted that to implement necessary changes in how individuals with disabilities access oral health care, members of the disability communities themselves should be invited to the conversation to communicate the challenges they face in obtaining optimal care to the political decision-makers that represent them.

Many nongovernmental organizations are now actively promoting an agenda that includes persons with disabilities [11, 12]. Educators are sharing best practice routines, and dental schools in the USA now have a stated requirement that students be competent in caring for persons with disabilities. The next task for educators is to begin to collect data from the disability communities themselves regarding lived experiences in seeking and receiving care. Efforts should be made to include the experiences of persons with disabilities in a variety of countries so that those solutions proposed correlate to the capacity of the health system in that particular location. Speakers acknowledged a lack of awareness among policy makers about the extent of the disenfranchisement of persons with disabilities from health and oral health services and even a lack of knowledge within the communities themselves about what should be a fundamental right and not a privilege. Here again, health literacy promotion should be a priority for both the provider and policy sectors. Opportunities for cross-disciplinary research and dialogue should be pursued so that the relationship between oral and general health is better understood by all sectors.

## Sustainable Development Goals and universal health care

The 74th World Health Assembly adopted a historic resolution [5] on oral health in 2021 that called for a paradigm shift in oral health policy and planning [13], away from a conventional model of restorative dentistry towards a promotive and preventive mode. Furthermore, it suggests that oral health be fully embedded into the noncommunicable disease (NCD) agenda, and that essential oral health interventions be included in universal health coverage (UHC) packages [5]. The proposed “common risk factor approach” discussed in the report recognizes, for instance, that NCDs and oral diseases share a set of key modifiable risk factors. High sugar intake, all forms of tobacco, and harmful alcohol use are major public health challenges for a wide range of NCDs and are also key modifiable risk factors for oral diseases.

Important questions remain: Is UHC realistic? Do the resources exist to achieve UHC anywhere in the world, let alone in low-income areas? There is reason for some optimism. An invited speaker, Steven Beshear, former governor of the state of Kentucky in the USA, described his efforts in bringing the Affordable Care Act (ACA), a policy initiative of a Democratic US president, to the majority Republican state of Kentucky. Kentucky narrowly approved participating in the ACA and expanded health coverage for low-income residents [14]. This coverage included expanding oral health benefits. A dramatic shift in health outcomes followed, and today, there is ample evidence that the ACA coverage not only saved the state money; it contributed positively to the economy by way of a healthier workforce. The state continues to participate in the ACA. Forum participants pointed to the need to assemble similar experiences and to gather more quantitative and qualitative data to justify the call for UHC and link it to favorable economic and societal progress. They advocated including more funding and attention to implementation science projects and greater effort to inform the public and policy makers about the value of investing in better oral health.

Lessons from the recent COVID-19 pandemic underscore the need for strong health systems accessible by entire populations, not just those with insurance coverage. There is also the opportunity to document the essential role played by members of the oral health workforce in safely delivering care in nonemergency care settings. The pandemic also had a catalytic impact on technologies like teledentistry, which can help bridge the gaps in services in rural communities or health professional shortages areas. A silver lining of the global shut down of dentistry was an explosion in new online learning materials as education moved from in-person to digital formats. These digital resources are enduring resources that are helping to unite the oral health community around the world to collaborate on issues of access, environmental best practice, and interprofessional practice.

There was frank discussion about historical resistance to workforce changes that emanate from the dental profession itself. The dental school curriculum leaves little room for study of policy, but improvements to the care system cannot occur without the involvement of the principal participants — dentists, hygienists, assistants, physicians, nurses, and the public at large. There is an enormous opportunity to educate a wider audience about how and where oral health is central not only to goal 3 (good health and wellbeing) of the UN Sustainable Development Goals (SDGs). In fact, oral health for all is relevant to many other of the 17 SDGs, from employment to decreasing poverty, to achieving a sustainable environment [15]. Oral health must emerge from its self-imposed silo to have a voice in and to advance each of these discussions.

## Research and education

As part of the US National Institutes of Health (NIH), the mission of the National Institute of Dental and Craniofacial Research [16] is to advance fundamental knowledge about dental, oral, and craniofacial health and disease and translate these findings into prevention, early detection, and treatment strategies that improve overall health for all individuals and communities across the lifespan. This agency is the principal government funder of oral research in the USA, yet it is one of the smallest of the 27 institutes and centers (US $475 million per annum, of which 60% is awarded outside dentistry) within the NIH, which had an annual budget of US $47.5 billion dollars in 2023. Add to this the equally small community of oral health researchers in the USA and around the globe, and it is clear that there is not a moment or penny to spare in generating quality science that can advance the agenda of oral health for all.

Participants in the forum referenced the need for dental schools to encourage high-performing students to consider research careers, to develop attractive career ladders, and to foster international exchange. There is a need to strengthen research capacity in lesser resourced countries so that relevant data can be collected to guide oral health policy. It is also obvious that oral health solutions will not be found within dentistry alone but will require collaboration among all of the health professions as well as with engineering, social science, and business, within a lens of diversity, equity, inclusion, and belonging. Investment in projects that enhance surveillance and health information systems are essential to provide decision support and relevant feedback for evidenced informed policymaking.

Within dental education, speakers pointed to a need to instill critical thinking from the predoctoral level on and to strengthen knowledge of how to interpret and apply data. There was a call to ensure that the dental curriculum has an equilibrium between practical research and theory.

An important task is to foster lifelong learners and to stay connected with providers after they graduate to foster adoption of new information. There was a call for recognition of the importance of industry being involved in mentoring and growing the research community. This industry partnership may be in the form of fellowships and sponsored research or through the creation of career paths for new scientists. Wherever possible, the research community should share data to discourage expenditures on redundant inquiries and to ensure that research is targeted to advancing the research agenda of the WHO and other global bodies. Gaps in knowledge identified include epidemiology and health information systems for surveillance of oral conditions; collection, harmonization, and rigorous assessment of evidence for equity in prevention and treatment of oral conditions and (population level) strategies to deliver essential quality oral health care without financial hardship [6].

For the USA, cost of dental education has been a formidable obstacle to encouraging dentists to consider a research career. Government and industry investment are imperative to reducing student debt and to make careers in research, academia, or policy practical. Technical advice provided to lesser resourced countries on less invasive, environmentally friendly dentistry should also be a priority [17].

## Public health initiatives and workforce

There was unanimous consensus among participants that championing prevention was the most important of the public health initiatives within the oral health landscape. That said, there was also agreement that not enough data exists to map the global distribution of oral disease, nor to understand behaviors and attitudes that contribute to poor oral health. It was suggested that data to better inform the policy landscape could be secured by partnerships with employers, unions, managed care, NGOs, and government agencies responsible for health and welfare at all levels.

A recent article notes that the levels and types of oral health problems occurring in populations change over time, while advances in technology change the way oral health problems are addressed and the way care is delivered [18]. Despite these facts, the methods used to plan the oral health workforce have remained rigid and isolated from planning of oral healthcare services and healthcare expenditure. The authors of this paper conclude that (a) workforce planning must be integrated with service delivery planning and the allocation of resources to oral health care, and (b) planning frameworks must be dynamic, responding to changes in population health and advances in oral health service delivery.

Forum participants strongly concurred and overlaid this dilemma with the divergence of experience in the global oral health workforce. This diversity ranges from countries in Africa in which there may be no dental schools, for instance, to countries like India in which the government, having assumed primary responsibility for public health, trains most of the country’s dentists, meaning every dentist is in fact a quasi-public health professional. One solution proposed was to develop and evaluate evidence-based workforce models specific to regional contexts and determine their effectiveness in improving leadership. Another proposed solution was to identify and incorporate unconventional workforce models from other disciplines such as nursing, including use of provider partners like community health workers and mid-level dental providers working with the supervision of dentists, or independently according to an officially approved scope of practice [15, 19]. Taken to its furthest extent, this model may even include caregivers for persons with disabilities who might be enlisted to administer disease preventive tools like fluoride varnish to arrest caries and minimize visits to the dentist.

## Action items

Based on the forum’s topics and discussions, the following series of action items were proposed:

### Equity and disability

#### Focusing on disability, equity, inclusion, and belonging

Focus on the inclusion of people with disabilities and other marginalized voices in places where decisions are made about oral health care.

- Develop and implement specific solutions at the regional level to improve the inclusion of people with disabilities and marginalized voices in decision-making related to oral health care.
- Encourage greater participation and collaboration among professionals from diverse backgrounds, including those outside of traditional oral healthcare fields, to address issues of equity.

#### Improving compensation models

Improve compensation models for reimbursement of oral health care.

- Shift the focus away from the purely economic aspects of oral healthcare practice models when considering compensation and reimbursement, with a view to promoting greater equity.
- Develop initiatives to increase health literacy and awareness among marginalized populations and oral healthcare professionals, with the goal of improving access to and quality of care and reducing disparities.

### Sustainable development goals and universal health coverage

#### Empowering local communities for sustainable oral health care

Incorporate a model of “speaking global and acting local.”

- Prioritize and tackle simple and achievable tasks related to oral health care at the local and regional levels, in alignment with the SDGs and UHC principles.
- Use community involvement and participation to build momentum and support for larger-scale policy changes in oral health care, based on the successes and insights gained at the local and regional levels.

#### Improving health literacy

Improve health literacy among people, political decision-makers, and all healthcare professionals.

- Take action to improve toolkits available to political decision-makers, informed by the perspective of oral healthcare professionals.
- Encourage oral healthcare professionals to expand their understanding beyond clinical practice and engage with the process of policy development.
- Focus on increasing knowledge and awareness of the interdependence and interconnectedness between all SDGs and their relationship to oral health.

#### Building learning health systems

Build continually learning health systems that are conducive to action.

- Integrate objectives of implementation science and scope of research.
- Implement grassroots efforts globally, adapting to each region’s specific needs and resources.

### Research and education

#### Oral health research agenda

Formulate an oral health research agenda with a global focus.

- Incorporate values and constructs of research guidelines and align with objectives of the WHO action plan in creating an oral health research agenda.

#### Scientific literacy

Increase scientific literacy to promote global adaptability.

- Conduct a thorough review of existing oral health curricula to identify areas that can be enhanced to encourage global thinking and scientific literacy.

#### Mentorship and leadership

Prioritize mentorship in research and provide guidance and leadership opportunities for students.

- Enforce equitable collaborative partnerships between stakeholders.
- Provide education grants for students to participate in research.

#### Research team composition

Improve and broaden research with an emphasis on diversity and inclusivity among research teams and partners.

- Offer more scholarships and loan repayment opportunities for students at an early stage of their career.

#### Convey scientific value

Convey the mission and vision of science by engaging with the public.

- Create awareness among the populations and encourage their participation in research.

#### Education costs

Devise plans to reduce the costs of education.

- Provide scholarships, funding opportunities, and incentives to work for the public good.

### Public health initiatives and workforce

#### Preventative approach

Focus on preventive oral health care.

- Create awareness of the impact and importance of oral disease prevention through education.
- Map the distribution of oral disease to prioritize oral health and hygiene needs.
- Understand behavioral changes and attitudes.
- Gauge current access to preventive healthcare.

#### Community health approach

Incorporate community public health models.

- Include community settings and worksites at large, and not only educational settings to provide broader access to public health interventions.

#### Transdisciplinary approach

Enhance partnerships with different disciplines to generate public policy.

- Engage with nontraditional oral health care professionals, such as social workers, nonacademic educators, religious and community leaders, and local politicians.

#### Leadership

Emphasize the importance of political leadership.

- Educate and train oral healthcare professionals in political advocacy and local, national, and global leadership.

#### Workforce models

Usage of nontraditional workforce models.

- Identify, educate, train, and incorporate nontraditional health providers from other disciplines such as nursing, educational staff, and community health workers.
- Develop and evaluate evidence-based workforce models specific to regional contexts and determine their effectiveness in improving health outcomes.
- Promote interprofessional and transdisciplinary learning and integrated planning.
- Conclusion
- Oral diseases affect billions of people globally and represent a pressing health concern that demands cohesive action. The Global Oral Health Forum underscored the urgency to break down silos and champion a unified strategy across stakeholders. Focusing on preventing rather than treating already existing oral diseases will positively affect equity, inclusivity, and care all throughout the lifespan, including for people with disabilities, will increase accessing oral health care, and reducing the burden of oral diseases globally. Key elements for success include fostering an all-stakeholders leadership approach that speaks with a unified voice and adopting a transdisciplinary approach to health care. The proposed action items represent an effort to ensure a holistic, comprehensive road map to improve oral health outcomes worldwide.

## Supplementary Information

**Additional file 1.** List of speakers and panelists for each session of the Global Oral Health Forum I.

## Data Availability

Not applicable.

## Abbreviations

ACA: Affordable Care Act
NCD: Noncommunicable disease
SDG: Sustainable Development Goals
UHC: Universal health coverage
UN: United Nation
WHO: World Health Organization
