# Supplementary material for: A unified voice to drive global improvements in oral health
Source: BMC Glob Public Health. 2023 Oct 1;1:19. doi: 10.1186/s44263-023-00019-0 (PMC11622904; doi:10.1186/s44263-023-00019-0)
Supplement: Supplementary file 1 — Additional file 1. List of speakers and panelists for each session of the Global Oral Health Forum I. [file 44263_2023_19_MOESM1_ESM.docx]

Additional file 1

| List of speakers and panelists for each session | |
| --- | --- |
| Sir Michael Marmot (UK) | Opening remarks |

| Session 1 - **Equity and disability** | | |
| --- | --- | --- |
| Speakers | Panelists | |
| Dr. Lois Cohen (USA)  Mr. John Kemp (USA)  Dr. Myechia Minter-Jordan (USA)  Dr. Mark Wolff (USA) | Dr. Laura Acosta (Mexico)  Dr. Ihsane Ben Yahya (Morocco)  Dr. Habib Benzian (Germany/South Africa)  Governor Steven Beshear (USA)  Dr. Stefan Listl (The Netherlands)  Dr. Rolando Peniche Marcin (Mexico)  Dr. Lena Natapov (Israel)  Ms. Allison Neale (USA)  Dr. Brian O’Connell (Ireland) | Dr. Garry Rayant (USA)  Dr. Francisco Javier Marichi Rodriguez (Mexico)  Dr. Maria Ryan (USA)  Mr. Mirco Stiehle (USA)  Dr. Mahesh Verma (India)  Dr. Deborah Weisfuse (USA)  Mr. Bryant Welters (USA)  Dr. Karen P. West (USA) |

| Session 2 - **UN Sustainable Development Goals and Universal Health Coverage** | | |
| --- | --- | --- |
| Speakers | Panelists | |
| Dr. Habib Benzian (Germany/South Africa)  Governor Steven Beshear (USA)  Dr. Stefan Listl (Netherlands) | Dr. Laura Acosta (Mexico)  Dr. Ihsane Ben Yahya (Morocco)  Dr. Lois Cohen (USA)  Mr. John Kemp (USA)  Dr. Rolando Peniche Marcin (Mexico)  Dr. Myechia Minter-Jordan (USA)  Dr. Lena Natapov (Israel)  Ms. Allison Neale (USA)  Dr. Brian O’Connell (Ireland) | Dr. Garry Rayant (USA)  Dr. Francisco Javier Marichi Rodriguez (Mexico)  Dr. Maria Ryan (USA)  Mr. Mirco Stiehle (USA)  Dr. Mahesh Verma (India)  Dr. Deborah Weisfuse (USA)  Mr. Bryant Welters (USA)  Dr. Karen P. West (USA)  Dr. Mark Wolff (USA) |

| Session 3 - **Research and Education** | | |
| --- | --- | --- |
| Speakers | Panelists | |
| Dr. Francisco Javier Marichi Rodriguez (Mexico)  Dr. Brian O’Connell (Ireland)  Dr. Mahesh Verma (India)  Dr. Karen P. West (USA) | Dr. Laura Acosta (Mexico)  Dr. Ihsane Ben Yahya (Morocco)  Dr. Habib Benzian (Germany/South Africa)  Governor Steven Beshear (USA)  Dr. Lois Cohen (USA)  Mr. John Kemp (USA)  Dr. Stefan Listl (Netherlands)  Dr. Myechia Minter-Jordan (USA)  Dr. Lena Natapov (Israel) | Ms. Allison Neale (USA)  Dr. Rolando Peniche Marcin (Mexico)  Dr. Garry Rayant (USA)  Dr. Maria Ryan (USA)  Mr. Mirco Stiehle (USA)  Dr. Deborah Weisfuse (USA)  Mr. Bryant Welters (USA) Dr. Mark Wolff (USA) |

| Session 4 - **Public Health Initiatives and Workforce** | | |
| --- | --- | --- |
| Speakers | Panelists | |
| Dr. Ihsane Ben Yahya (Morocco)  Dr. Lena Natapov (Israel)  Dr. Maria Ryan (USA)  Mr. Bryant Welters (USA) | Dr. Laura Acosta (Mexico)  Dr. Habib Benzian (Germany/South Africa)  Governor Steven Beshear (USA)  Dr. Lois Cohen (USA)  Mr. John Kemp (USA)  Dr. Stefan Listl (Netherlands)  Dr. Rolando Peniche Marcin (Mexico)  Dr. Myechia Minter-Jordan (USA)  Dr. Brian O’Connell (Ireland) | Dr. Garry Rayant (USA)  Dr. Francisco Javier Marichi Rodriguez (Mexico)  Mr. Mirco Stiehle (USA)  Dr. Deborah Weisfuse (USA)  Dr. Mahesh Verma (India)  Dr. Karen P. West (USA)  Dr. Mark Wolff (USA) |
